# EVALUATION OF THE THORACOABDOMINAL MOBILITY OF OBESE SUBJECTS IN PRE-BARIATRIC SURGERY

**DOI:** 10.1590/0102-6720201600S10011

**Published:** 2016

**Authors:** Ana Paula Limongi Richardelli VELOSO, Karla Garcez CUSMANICH

**Affiliations:** University of Taubaté (UNITAU) and Vida Vale Clinic, Taubaté, SP, Brazil

**Keywords:** Abdominal circumference, Obesity, Physical therapy specialty, Bariatric surgery

## Abstract

**Background:**

Obesity can affect the thorax, diaphragm, and alterations in respiratory function even if the lungs are within normality. The respiratory compliance is very reduced by the increase in fat mass.

**Aim:**

To evaluate the effect of the physical therapeutic respiratory exercises on the thoracoabdominal mobility of obese individuals in pre-bariatric surgery

**Methods:**

Cross-sectional and descriptive study, which used the cirtometry (axillary, xiphoid and abdominal) to evaluate the mobility of 74 individuals, 27 men and 47 women, in pre-bariatric surgery, assisted by the team EMAD, after eight weeks of physiotherapy, following a protocol of exercises, reevaluating and compared the measures pre and post intervention.

**Results:**

Had positive correlation abdominal mobility in the total volume of all participants (p=0.010) and also for all the measures in the measurement of residual volume in three levels (p=0.000). Comparing genders, in total volume, cirtometry abdominal greater for women (p=0.015) when compared to men and residual volume, significance for either men or women in all measurements (p=0.000).

**Conclusion:**

Obese patients that underwent the physiotherapeutic treatment during the preoperative period, had pré respiratory dynamics improved by the increase in the mobility of the chest cavity and by the improvement of respiratory conscience.

## RESUMO

**Racional:**

A obesidade pode afetar o tórax e o diafragma, determinando alterações na função respiratória, mesmo quando os pulmões se apresentam dentro da normalidade. A complacência respiratória é muito reduzida pelo aumento de massa gordurosa.

**Objetivo:**

Avaliar o efeito de exercícios fisioterapêuticos respiratórios sobre a mobilidade torácica de indivíduos obesos no pré-operatório de cirurgia bariátrica.

**Método:**

Estudo transversal e descritivo, que utilizou a cirtometria (axilar, xifoide e abdominal) para avaliar a mobilidade de 74 indivíduos, 27 homens e 47 mulheres, em pré-operatório de cirurgia bariátrica, assistidos pela equipe EMAD, após oito semanas de fisioterapia, seguindo um protocolo de exercícios, reavaliando e comparado as medidas pré e pós a intervenção.

**Resultado:**

Apresentaram correlação positiva a mobilidade abdominal no volume total de todos os participantes (p=0,010) e também para todas as medidas na mensuração do volume residual nos três níveis (p=0,000). Comparando os sexos, no volume total, a cirtometria abdominal maior das mulheres (p=0,015) quando comparada a dos homens e no volume residual, significância tanto para os homens quanto para as mulheres em todas as mensurações (p=0,000).

**Conclusão:**

Obesos que se submeteram ao tratamento fisioterapêutico durante o período pré-operatório, tiveram dinâmica respiratória melhorada pelo aumento da mobilidade da caixa torácica e pela melhora da consciência respiratória.

## INTRODUCTION

Candidates for bariatric surgery are patients with BMI greater than 40 kg/m^2^ or greater than 35 kg/m^2^ associated with comorbidities^3^ ^,^ ^7^. Abdominal surgeries can affect the respiratory musculature by means of different mechanisms, such as pain and the loss of the integrity of the abdominal muscles by incision and neuromuscular blockers for anesthesia, which interfere in the muscle contractility, and contribute to inadequate performance of respiratory muscles after operation^4^ ^,^ ^15^​.

General anesthesia has been cited as an important risk factor for respiratory changes in the postoperative period. May be related to the presence of bronchospasm by trauma to the airway, decreased pulmonary compliance by changing the distensibility of the lungs, reduction of chest expansion, early closing of the airways and ventilation redistribution for the zones, potential for superior pulmonary atelectasis and postoperative hypoxemia^8^ ^,^ ^16^. The majority of the postoperative complications arise from preoperative causes and some exercises can be made to decrease the chances of complications that end up increasing the hospital stay^1^ ^,^ ^14^.

In obese the diaphragm is located in upper position, which may decrease functional residual capacity^9^. Obesity typically promotes changes in respiratory function, by presenting a large quantity of adipose tissue around the ribcage (decreasing complacency), and also by the elevation of the diaphragm, caused by compression made by abdominal content. These factors lead to decreased functional residual capacity and alter its gaseous exchanges, in virtue of the superficial ventilation^2^ ^,^ ^13^. The complacency of the respiratory system is very low, because of the increase in mass of the chest wall and limited diaphragmatic excursion^17^ ^,^ ^19^.​

Studies^9,17^ performed in obese not diagnosed with other diseases have suggested that pulmonary and chest wall compliance decrease due to fat deposition in the chest and abdomen, fact that leads to increased elastic retraction and reduced distensibility of extra pulmonary structures. Obesity can affect the thorax, diaphragm, and alterations in respiratory function even if the lungs are within normality^22^.​These changes are due to increased respiratory effort and impairment of the gas transport system. The hypertonia in the abdominal muscles, is another symptom associated to obesity, which compromises the respiratory function dependent on the role of the diaphragm^6^ ^,^ ^22^.​ For the purposes of respiratory muscle training and development of habit to recovery postoperative pulmonary, some respiratory actions are adopted, among them, the use of inspiratory stimulants. Normally patients are advised to train before operation, in series, some times a day, throughout the pre-operative period.​ This modality of physical respiratory training is intensified in the moment of admission^14^ ^,^ ^21^.​ 

This study aimed to evaluate, through cirtometry, the effect of respiratory physical therapeutic exercises on the thoracoabdominal mobility of obese individuals in pre-bariatric surgery.​

## METHOD

Were selected 74 individuals in the pre-bariatric surgery. All belonged to the group of patients attended at the Vida Vale Clinic, Taubaté, SP, Brazil, which passed by monitoring of multiprofessional team counting with three surgeons, cardiologist, endocrinologist, psychologist, nutritionists and physiotherapy, in preparation for bariatric surgery. All participants signed a free and informed consent. This study with protocol number 1.188.024 was approved by the Ethics in Research.​

The only inclusion criterion was being obese in pre-bariatric surgery and the exclusion with BMI<30 kg/m^2^. ​

irtometry was used as measurement method and the measurements were performed with the volunteers on foot, in ground. 

To start the procedure learning maneuver was made to maximum inspiration and after maximum expiration, measuring the three regions: 1) axillary perimeter with a metric tape passing by axillary cavus; 2) xiphoid perimeter, passing on the xiphoid appendix at the level of the 7^th^ costal cartilage; and 3) abdominal perimeter, passing through the umbilicus. Three measurements were carried out by noting the best value (greater difference between measures), maximum inspiration (total lung capacity - TLC) assuming a forced expiration and maximum expiration (residual volume - RV) starting from the deep inspiration.​

**FIGURE 1:**
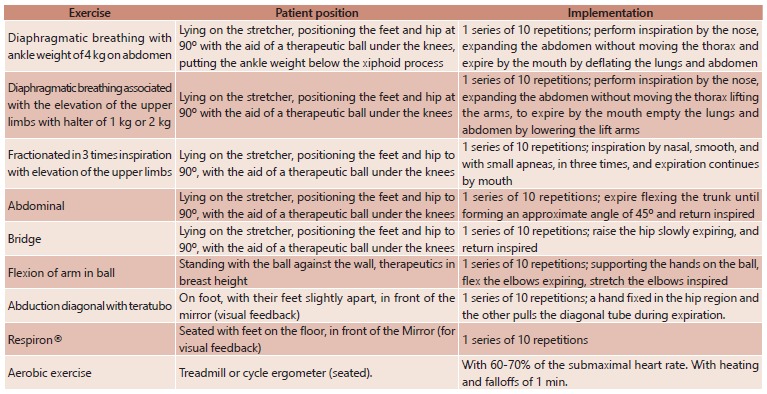
Exercise protocol

After the initial assessment the results were recorded on record for each volunteer and all guidelines were carried out, including the purchase of incentive spirometry device. Afterwards, the second session began with the exercise protocol consisted in physiotherapeutic exercises for breathing consciousness, strengthening upper limbs, trunk and aerobic during eight weeks and, after completed this phase, another cirtometry was made for comparing the values. The program consisted of eight sessions of physical therapy once a week, being initially collected vital signs (heart rate, respiratory rate, oxygen saturation, blood pressure and pulmonary auscultation) and then performing the exercises described in Figure 1.

 Was orientated to all patients to perform at home a series of 10 repetitions of Respiron^(r)^ at least once a day, plus diaphragmatic breathing and a series of 10 repetitions whenever remembered during the day​.

### Statistical analysis

The data were analyzed using the Stata version 11.0 and treated with descriptive analysis (mean and standard deviation) and inferential. The Shapiro-Wilk test was used to verify the normality of the data and homogeneity of variance. For the comparison between initial and final measurements obtained by cirtometry, was used the paired t-test. The level of significance adopted was 5% (p<0,05). 

## RESULTS

###  Participants profile 

 Seventy-four volunteers between 17-70 years of age were enrolled, with an overall mean of 37.4+/- 11.1 years; 47 were women with a mean of 37.9+/-11.2 years; and 27 men with average of 37.1 (±11.0). 

 The general mean BMI was 41.7+/-4.8 kg/m^2^ with variation of 34.1-57.1 kg/m^2^. Regarding the classification of obesity according to the BMI, the majority (56.2%) was classified as grade III; 42.5% degree II; and 1.3% grade I.

 The distribution of BMI according to gender showed that the category most prevalent among women was obesity II (53.2%) and among men the obesity III (76.9%).

###  Thoracoabdominal mobility - total volume 

 The values found are described in Table 1. There was no significant difference in axillary and xiphoid mobility among measurements obtained before and after the application of the exercises. Already the abdominal mobility was significantly higher in comparison to the obtained before the exercises. 

**TABLE 1:** Thoracoabdominal mobility and total volume evaluation values in pre- and post-respiratory therapy

Mobility(cm) Total volume	Initial	End	p
Axillary	120,3 ± 10,5	119,7 ± 10,1	0.072
Xiphoid	113,6± 10,1	113,9± 10,0	0.844
Abdominal	123,0± 12,3	124,3 ± 11,8	0.010*

The data are expressed in mean±standard deviation, * statistical significance (p<0.05).

​ Thoracoabdominal mobility and total volume evaluation values in pre- and post-respiratory therapy showed a positive correlation only for the abdominal mobility (p=0.010). As to gender, it was observed greater significance for abdominal cirtometry in women when compared to men (Table 2).

**TABLE 2:** Thoracoabdominal mobility and total volume evaluation values according to gender in pre- and post-respiratory therapy

Mobility (cm) Total Volume	Initial		End		P Value	
	Male	Female	Male	Female	Male	Female
Axillary	129,8 ± 7,5	114,8 ± 7,6	128,7 ± 7,6	114,4 ± 7,2	0,048*	0,365
Xiphoid	121,7± 7,7	109,0±8,3	121,9± 7,3	109,2± 8,2	0.706	0,890
Abdominal	133,0± 11,0	117,3± 9,0	134,3 ± 11,0	119,1± 8,8	0.525	0,015*

The data are expressed in mean ± standard deviation, * statistical significance (p<0.05).

###  Thoracoabdominal mobility - residual volume 

​ The values found are described in Table 3. The axillary, xiphoid and abdominal postoperative mobility after physical therapy were significantly higher in comparison to the measured initially. In all measures there was reduction of cirtometry. 

**TABLE 3:** Thoracoabdominal mobility and residual volume evaluation values in pre- and post-respiratory therapy

Mobility (cm) Residual volume	Initial	End	p Value
Axillary	117,3 ± 10,5	115,0 ± 10,3	0.000*
Xiphoid	112,4± 10,2	109,4± 9,8	0.000*
Abdominal	124,1 ± 12,2	118,9 ± 11,9	0.000*

The data are expressed in mean ± standard deviation, * statistical significance (p<0,05).

**FIGURE 2:**
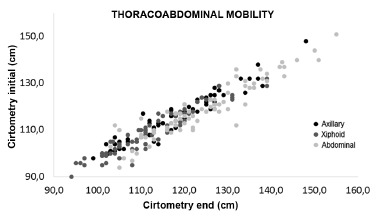
Dispersal diagram of the thoracoabdominal mobility and residual volume

Figure 2 shows the dispersion diagram of the data of the thoracoabdominal mobility and residual volume before and after respiratory physiotherapy. The values found showed positive correlation for all measures (p=0.000).

Table 4 demonstrates the mobility regarding gender, and can be observed significance to both men and women in all measurements. 

**TABLE 4:** Thoracoabdominal mobility and residual volume values according to gender in the pre- and post-respiratory physiotherapy

Mobility (cm) Total volume	Initial		End		p	
	Male	Female	Male	Female	Male	Female
Axillary	126.7±7.6	111.9±7.7	123.8±8.7	109.9±7.3	0.000*	0.000*
Xiphoid	120.1±7.8	107.9±8.7	117.4±7.1	104.8± 8.1	0.000*	0.000*
Abdominal	133.8±10.8	118.5± 9.1	127.9±11.3	113.7± 8.9	0.000*	0.000*

The data are expressed in mean±standard deviation; * statistical significance (p<0).

## DISCUSSION

Several studies confirm that obesity has a series of different corruptive effects on respiratory function and may be total factor capable of potentiating the development of pulmonary complications.

In obesity, alterations in respiratory function more frequently found are the reduction of expiratory reserve volume and the functional residual capacity, because of the changes in chest wall mechanics, decreasing total respiratory compliance, of the pulmonary volume, reduction in residual volume and its relation with the total lung capacity^22^.​

Forti et al^.10^ mentioned that the bariatric surgery may lead to changes in the respiratory mechanics and pulmonary function. Thus it is of great importance to achieve adequate respiratory evaluation, both at the pre- and postoperative period, with views on the actuation of respiratory therapy in the prevention and rehabilitation of these patients. As already known, surgical incision in the abdomen and ribcage, can affect the integrity of the respiratory muscles directly affecting its function. The weakening of the respiratory muscles after operation can lead to postoperative complications impairing respiratory function and increase the hospital stay^12^. For these reasons, this paper aimed to minimize these respiratory complications. 

These results showed that the measures have improved significantly after the implementation of the exercise protocol. However, one of the main findings was that none of the patients assisted had respiratory complication after surgery. 

Paulin et al.^18^ performed a study with 15 patients showing that exercises directed to the increase the mobility of the chest cavity improves chest expansion, the quality of life and the submaximal exercise capacity. As mentioned above, it was evidenced here that exercises directed to the respiratory musculature not only have been able to increase the mobility and expandability, as also the consciousness, improving respiratory implementation of diaphragmatic muscles, increasing the thoracoabdominal mobility; this was more evident in the abdominal region, when compared to the axillary and subxiphoid in both genders, mainly in the cirtometry measurement of residual volume. 

One study performed in 2011 found the efficacy of pulmonary rehabilitation program to promote significant alterations in respiratory mechanics of 20 obese women, indicating an improvement of thoracic mobility^20^, similar to the present study; however, here were evaluated women and men and also compared the values between the genders. 

Results obtained with this treatment program have a relation with the result presented in the study with obese patients of Costa et al.^5^, where it was concluded that respiratory retraining functional applied to seven patients, promoted changes in respiratory mechanics, more properly the thoracoabdominal mechanics. The amplitude levels to axillary and abdominal ones, suggested that these individuals may have their pulmonary function altered through the functional respiratory retraining. These changes promoted inspiratory muscle strength and increased abdominal mobility preventing complications.

​ The physiotherapeutic treatment has important role during the pre- and postoperative period in the bariatric surgery^10^ ^,^ ^11^. ​It is a consensus that the physiotherapeutic treatment during the preoperative period, when indicated as an adjuvant for surgical preparation, represents an important tool in the reduction of complications related to extubation difficulties and atelectasis, among others.

 It is expected that the increase in respiratory conscience, in addition to providing the best conditions for which the patients face the operation, can also be useful in the early recovery in the postoperative period. 

​ No papers were found correlating the deficit of respiratory conscience, justifying the muscle strength and mobility found in the great majority of obese. 

​ The exercise program addressed in this study, aiming at the increase in the mobility of the chest cavity, improved it in all levels, proven by values obtained on cirtometry. This can represent one more important tool in the approach of pulmonary rehabilitation, aiming a better quality of life and, in this case, the reduction of postoperative complications favoring shorter hospitalization time, and it´s known benefits. 

​ 

## CONCLUSION

 Obese patients that underwent the physiotherapeutic treatment during the preoperative period, had respiratory dynamics improved by the increase in the mobility of the chest cavity, and also by the improvement on respiratory conscience. 
